# Risk factors and short-term respiratory outcomes associated with unplanned extubations during neonatal intensive care

**DOI:** 10.3389/fped.2025.1643057

**Published:** 2025-08-12

**Authors:** Irma Mannerstedt, Laszlo Markasz, Victoria Karlsson, Nils Pettersson, Ylva Thernström Blomqvist, Johan Ågren, Richard Sindelar

**Affiliations:** ^1^ Department of Women’s and Children’s Health, Uppsala University, Uppsala, Sweden; ^2^ Department of Electronic Patient Journals, Uppsala University Hospital, Uppsala, Sweden

**Keywords:** unplanned extubation, neonate, preterm infant, skin-to-skin care, neonatal intensive care, mechanical ventilation

## Abstract

**Background:**

Unplanned extubation (UE) represents an unwanted adverse event in neonatal intensive care. Although skin-to-skin care (SSC) in intubated infants receiving mechanical ventilation (MV) is challenging, its impact on the incidence of UEs has not been reported.

**Purpose:**

To determine the incidence, infant characteristics, and short-term respiratory outcomes of UEs in a unit applying SSC as standard of care also during MV.

**Methods:**

Single-center retrospective cohort study including all infants receiving MV in a Swedish tertiary care center during 2021–2023. UE incidence was calculated per 100 days of MV related to time spent in conventional care (CC) and SSC, using automated chart review of electronic medical records. Pre-defined short-term respiratory outcomes were mode of respiratory support, ventilator settings and fraction of inspired oxygen (FiO_2_), at 30–120 min post-UE.

**Results:**

The UE incidence was 3.9 per 100 days of MV (3.0 in CC vs. 10.4 in SSC; *p* < 0.001). The UE incidence during SSC decreased from 14.5 in 2021, to 7.7 in 2023 (*p* = 0.07), whereas it remained the same during CC. After UE, 72% infants were reintubated within 120 min, and showed an increased mean FiO_2_ (0.37 vs. 0.43; *p* = 0.01).

**Conclusions:**

The number of UEs were high during SSC but decreased during the study period. Reintubation was not required in >25% of all UEs, regardless of type of care. Following UE, an increased need for supplemental oxygen was observed. Safe SSC in mechanically ventilated infants requires experienced staff and increased staff and parental risk awareness.

## Introduction

Unplanned extubation (UE), i.e., the unintended dislodgement of the endotracheal tube (ETT) (1) is a serious adverse event in neonatal intensive care with complications such as bronchospasm, bradycardia, and prolonged subsequent mechanical ventilation (2). The emergency reintubations that often follow has been associated with both a higher number of intubation attempts, and a higher rate of airway injury (3).

Compared to the pediatric and adult intensive care populations, UE is more frequently observed in neonates (4), likely due to a less frequent use of sedation (5), shorter trachea, and the use of uncuffed ETTs. Accordingly, reported risk factors for UE include infant agitation, poor ETT fixation, bedside procedures (e.g., suctioning or re-taping of the ETT) (2), and high ETT positions (6). A review by Silva et al. report a median incidence of 1.98 (IQR: 0.14–5.3) per 100 ventilator days (2).

Skin-to-skin care (SSC) of preterm infants is a mode of care with several important advantages for both the infant and parents. Compared to conventional care (CC) in a crib/incubator SSC has been shown to reduce mortality (7), lessen the risk of infection and hypothermia, improve physiological stability (8), and reduce pain response (9). While SSC is increasingly being applied also in a high-tech environment in the care of infants born extremely preterm (10), the impact of SSC on the incidence of UE risk has to our knowledge not been previously investigated.

We aimed to study the incidence of UEs in a setting where SSC is extensively applied also during mechanical ventilation, and compare the incidence of UEs during SSC and CC. We also aimed to evaluate the short-term respiratory outcomes after UE.

## Methods

### Subjects

All intubated and mechanically ventilated infants in the Neonatal Intensive Care Unit at the Uppsala University Children's Hospital in Sweden from January 2021 to December 2023 were included in this retrospective cohort study. All data were extracted from the electronic medical record (EMR) and Patient Data Management System (MetaVisionNICU, iMDSoft, Tel Aviv, Israel) directly from the database using algorithms created on demand queries and algorithms with a sampling rate of 5 per min and an average presented automatically every minute. This additional layer of quality assurance laid a solid foundation for the rest of the analyses. Data included infant demographics as well as details on each event regarding mode of care (CC or SSC), and respiratory mode (30-min before and 30–120-min post UE).

### Treatment of data and statistical analyses

UEs were defined as any unplanned dislodgement of an ETT. Each UE was considered a separate event thus including multiple events within the same episode of care in a number of infants. The incidences of UE in total, and by mode of care (CC and SSC) were calculated per 100 ventilator days. Outcomes after UE were defined by the type of respiratory support: non-invasive ventilatory assistance or reintubation with invasive mechanical ventilation; changes in FiO_2_ or ventilatory settings. Data are presented as median (IQR) and regression analyses performed to evaluate changes over time. Microsoft® Excel was used to process all data, perform calculations and statistical analysis. Student's two-tailed, heteroscedastic *t*-test was applied to test the significance between group means. Regression analysis was performed to find linear correlations, and Pearson analysis was applied to find the significance of these correlations. A *p* < 0.05 was considered statistically significant.

### Ethical permission

The study was conducted in accordance with the declaration of Helsinki. The study was approved by the Swedish Ethical Review Authority (D:nr 2024-03652-01).

## Results

**Figure 1:**
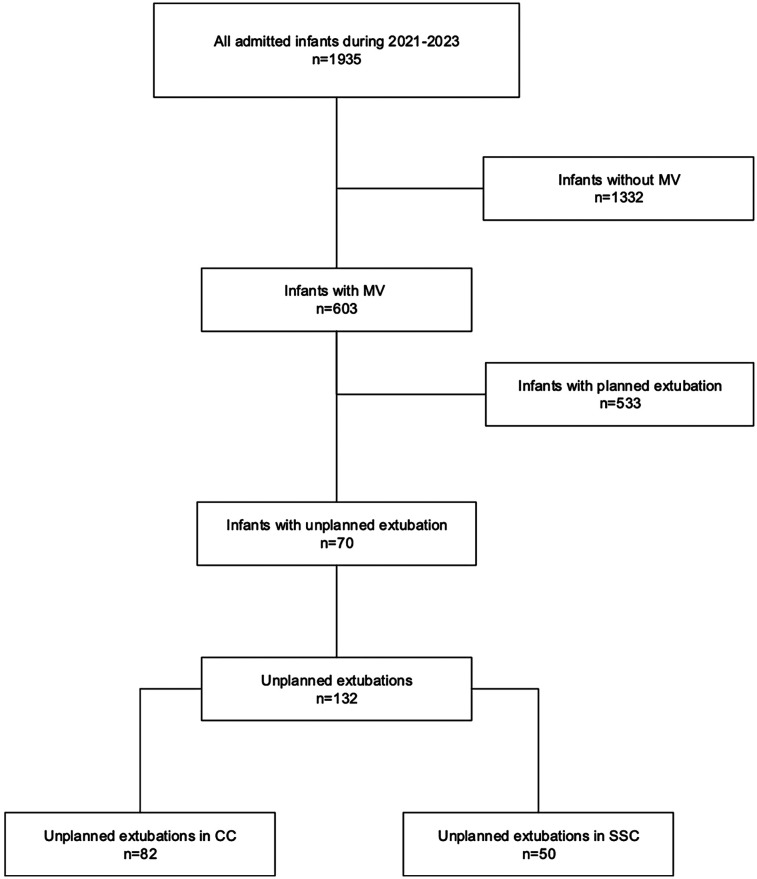
Flow chart of infants included in the study.

**Figure 2:**
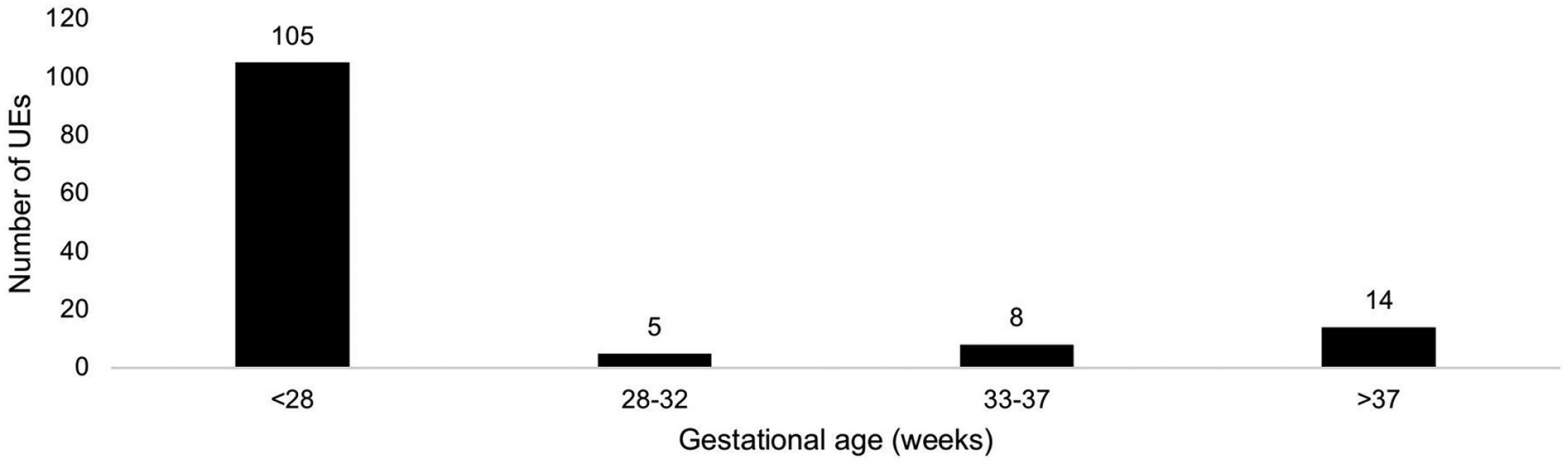
Number of unplanned extubations per gestational age (GA) category. Minimum GA 22^+0^; maximum GA 41^+2^. Note the higher incidence in infants born before 28 weeks GA.

Of all infants admitted during the study period 2021–2023 (*n* = 1935), 603 infants received mechanical ventilation for a total of 3,353 days (Figure 1). Sedatives and/or analgesia are not used routinely in infants receiving mechanical ventilation in our unit, but all infants are assessed regularly for pain and stress with observational pain scores (Neonatal Pain, Agitation and Sedation Scale) and vital signs, if need for sedation/analgesia would arise. The standardized route for intubation was the oral route (*n* = 128), four patients were nasally intubated. The total number of UEs during the study period was 132 in 70 patients. Among these 70 patients 38 had one UE, and 32 had more than one UE (maximum 7 UEs in one patient). Eighty-two (62%) and 50 (38%) of UEs occurred during CC and SSC, respectively. The infants were predominately born extremely preterm (<28 weeks gestation) (Figure 2), their demographical characteristics are displayed in Table 1. No difference in ventilatory settings or FiO_2_ were noted between CC and SSC prior to UE. The mean daily duration of SSC in all infants on mechanical ventilation during 2021–2023 and irrespective of UE, was 2.67 h (range 1.50–4.98 h).

**Table 1:** Characteristics of 2021–2023 cohort (*n* = 70) experiencing unplanned extubations.

Unplanned extubations (*n*)	132
Gestational age (weeks)	24^+2^ (22^+6^–26^+6^)
Birth weight (g)	660 (544–923)
Weight at UE (g)	844 (613–1,439)
Postmenstrual age at UE (weeks)	26^+3^ (25^+0^–29^+2^)
Postnatal age at UE (days)	14 (7–23)
Postnatal age at UE in CC (days)	15 (8–23)
Postnatal age at UE in SSC (days)	14 (7–20)
Time from intubation to UE (h)	47 (19–127)
Total time on MV (days)	3,353
Total time on MV in CC (days)	2,884 (86)
Total time on MV in SSC (days)	469 (14)

Data are median (IQR), or *n* (%). UE, unplanned extubation; MV, mechanical ventilation; CC, conventional care; SSC, skin-to-skin care.

### Incidence of unplanned extubation

**Figure 3:**
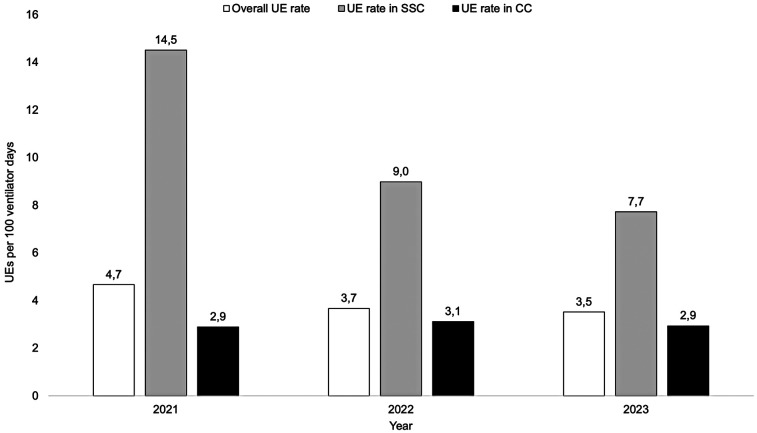
Incidence of unplanned extubations (UE) during 2021–2023. Note the decreasing incidence of UEs in skin-to-skin care (SSC) while the incidence in conventional care (CC) remained at similar levels.

The mean incidence of UEs during the study period was 3.9 UEs per 100 ventilator days. The incidence of UEs in SSC vs. CC were statistically different for the study period, being 10.4 in SSC, and 3.0 in CC (*p* < 0.001). The incidence of UE during SSC declined (*r* = −0.3; *p* = 0.07) while there was no change in the UE incidence during CC (*r* = 0.03; *p* = 0.85) from 2021 to 2023 (Figure 3). The mode of ventilation when UE occurred was A/C (*n* = 72), SIMV (*n* = 38), NAVA (*n* = 14) and HFOV (*n* = 8); no difference in mode of ventilation was observed between SSC vs. CC.

### Short term respiratory outcomes after unplanned extubation

After experiencing a UE, 89 out of 132 cases (67%) were reintubated within 30 min, increasing to 95 out of 132 (72%) within 120 min. Accordingly, almost a third of the infants (*n* = 37) were successfully transitioned to non-invasive respiratory support after UE, with no observed difference between SSC (*n* = 12) and CC (*n* = 25). Among those reintubated, no significant changes in airway pressures were noted however, the fraction of inspired oxygen (FiO_2_) increased from a mean of 0.37 (0.20 SD) pre-UE to 0.43 (0.20 SD) 120 min post-UE (*p* < 0.001). There were no differences in ventilatory settings or FiO_2_ when comparing SSC and CC. No death was associated closely to the time point of unplanned extubation.

## Discussion

The present study presents data on the incidence of UE in neonatal intensive care depending on whether the infant is receiving SSC or CC. In the studied cohort, UE was several-fold more frequent during SSC than CC. The overall incidence of UE per 100 ventilator days over the 3-year period was 3.9, comparable to previous studies. A review by Silva et al. reported a median incidence of 1.98 (IQR: 0.14–5.3) per 100 ventilator days (2), while others studies reported rates ranging from 2.1 (11) to 3.3 (12), and 4.75 (13). A benchmark target of less than 1 UE per 100 ventilator days has been suggested by Silva et al. (2), as a realistic aim of quality improvement. The infants in our cohort had lower gestational age at birth than in the studies mentioned previously (GA 24.3 vs. 27.3, 27, and 26.7) (11–13), which might have led to a longer time on mechanical ventilation and our relatively higher UE incidence.

To our knowledge, this is the first study reporting the UE incidence during different modes of care, together with the time spent in the respective modes. The two previous studies that have reported a lower proportion of UEs occurring during SSC (12, 14) than in our study, did not take into account the length of mode of care, making absolute comparisons with our study difficult. Clearly, an approach where infants are only briefly cared for in SSC will demonstrate a low proportion of UE occurring during this mode of care. For feasible future comparisons we would like to propose applying the number of UEs per 100 ventilator days as the preferred measurement.

Seventy-two percent of the patients required reintubation within 120 min after the event, and while previous studies have reported lower reintubation rates (2, 3, 15), those cohorts included infants with several weeks higher median gestational age which may have influenced the outcome. Given that reintubation was not required in 28% of events, this implies that these infants could have been extubated earlier, under controlled circumstances, suggesting an opportunity to improve the identification of extubation readiness. The reintubation rates and ventilatory settings were similar when comparing SSC and CC. Oxygen requirements were found to be slightly higher after UEs that required reintubation which further strengthens the need to avoid such a stressful and potentially harmful adverse event (16).

The use of uncuffed tubes and the low use of sedatives during prolonged MV may influence the high incidence of UEs in our NICU (5, 17). In the present study almost all ETTs were uncuffed and placed using the oro-tracheal route according to standardized routine. While it has been argued that the nasal route allows a more secure ETT fixation (17), it has not been demonstrated to affect the rates of UE (18). Previously reported risk factors for UE, including poor ETT fixation, bedside procedures (e.g., suctioning or retaping of the ETT) (2), and high ETT positions (6), are also potential areas to analyze to identify patterns of UEs, and thereby areas of improvement.

During the three-year study period, we observed a decreased incidence of UE during SSC. While we cannot demonstrate any cause-effect relationship given the observational nature of our investigation, a quality improvement (QI) initiative was launched in November 2021, consisting of monthly feed-back reporting of the UE incidence on a staff lunchroom information screen as well as raising awareness about these highly unwanted adverse events at repeated staff meetings. As previously documented (5) it is conceivable that this might have contributed to the observed decline in UE, particularly during SSC. Nevertheless, the high rate of UE during SSC is of concern and should be further targeted in future QI efforts.

We believe that the observed increased risk of UE during SSC should not be taken as an argument to withhold or postpone SSC, given its many advantages such as physiological stability (19). The postnatal age at UE did not differ in SSC and CC (14 vs. 15 days), implying similar clinical settings at UE. SSC was initiated after 4–6 days of life for 22–24 GA, after 1 day for 25 GA, after first hours of life for 25–26 GA, and immediately after birth for >26 GA in the present cohort. Rather than refraining from SSC, optimization of routines for ETT fixation and positioning of the ventilator tubing, transfer procedures, and raising staff and parental awareness should be further pursued in order to enable both a lowest possible rate of UE, and parent-infant closeness.

### Study limitations

The study has several limitations. The generalizability is limited by being from a single-center with an active strategy to provide early and prolonged SSC also in infants born extremely preterm. Thus, we did not have equipoise to randomize infants to one mode of care or the other. Still, by comparing the number of UE in relation to time spent in CC or SSC we believe that the finding of higher UE incidence in SSC than during CC to be reliable. Further, since the events are registered manually in the EMR by the nursing staff we cannot exclude a reporting bias for one mode or the other. The pragmatically chosen respiratory outcomes were short-term and limited by the increasing complexity of using data extraction algorithms to gather large quantity of data processable without high performance computing. It cannot be precluded that other follow-up intervals or longer-term outcomes would have yielded different results, but the observational nature of the study limits any such further analyses.

## Conclusions

In summary, the UE incidence was generally high and affected oxygen requirements after reintubation, further strengthening the adversity of the event. When correcting for time spent in each mode of care, expressed as UEs per 100 days of mechanical ventilation, the UE incidence was higher during skin-to-skin care than during conventional care. Future QI initiatives are necessary to enable both a low risk of unplanned extubation, and parent-infant closeness.

## Data Availability

The original contributions presented in the study are included in the article/Supplementary Material, further inquiries can be directed to the corresponding author.
